# Loss of *putzig* in the germline impedes germ cell development by inducing cell death and new niche like microenvironments

**DOI:** 10.1038/s41598-019-45655-5

**Published:** 2019-06-24

**Authors:** Ludmilla Kober, Mirjam Zimmermann, Michaela Kurz, Melanie Bayer, Anja C. Nagel

**Affiliations:** Institute of Genetics (240), University of Hohenheim, 70599 Stuttgart, Germany

**Keywords:** Stem-cell niche, Oogenesis

## Abstract

Germline stem cell development and differentiation is tightly controlled by the surrounding somatic cells of the stem cell niche. In *Drosophila* females, cells of the niche emit various signals including Dpp and Wg to balance stem cell renewal and differentiation. Here, we show that the gene *pzg* is autonomously required in cells of the germline to sustain the interplay between niche and stem cells. Loss of *pzg* impairs stem cell differentiation and provokes the death of cells in the germarium. As a consequence of *pzg* loss, increased growth signalling activity predominantly of Dpp and Wg/Wnt, was observed, eventually disrupting the balance of germ cell self-renewal and differentiation. Whereas in the soma, apoptosis-induced compensatory growth is well established, the induction of self-renewal signals during oogenesis cannot compensate for dying germ cells, albeit inducing a new niche-like microenvironment. Instead, they impair the further development of germ cells and cause in addition a forward and feedback loop of cell death.

## Introduction

Stem cells maintain tissue homeostasis by a continuous and strictly controlled process of self-renewal and differentiation. In contrast to somatic cells, germline stem cells give rise to gametes with the ability to establish an entire organism upon fertilization, thereby passing genetic and epigenetic information from one generation to the next. Intricate developmental processes ensure the self-renewal and correct cellular differentiation of germline cells for a continuous supply of gametes and the protection of genome integrity. *Drosophila* oogenesis is a well-established model system to study those regulatory processes that are likely to apply widely to other organisms.

The adult *Drosophila* ovary consists of individual units named ovarioles, which harbour progressively developed eggs (for review^1,2^). At the anterior tip of each ovariole, two to three germline stem cells (GSCs) reside in a structure called the germarium, where they are directly associated with cells from somatic origin comprising the stem cell niche^3,4^. The intimate contact of the GSC with the niche is key to its further development, allowing for an asymmetric division resulting in another GSC and a cystoblast. The cystoblast divides further to eventually give rise to a germline cyst including the oocyte^2^. The niche/GSC contacts are hence a strict requirement for self-renewal and subsequent differentiation of the GSC alike. The somatic niche includes the terminal filament cells and the underlying cap cells that direct the self-renewal capacity of GSCs^4–7^. Adhesion proteins DE-Cadherin and beta-catenin/Armadillo (Arm) mediate recruitment of GSCs to the niche and their anchorage to the cap cells. Accordingly, respective mutants affect GSCs maintenance^8,9^. Moreover, differing DE-Cadherin levels mediate GSC’s competition for niche contacts, resulting in the loss of some GSCs, perhaps serving as a quality control mechanism for removing e.g. precociously differentiated stem cells from the niche^10^. Besides this physical regulation of GSC self-renewal, a complex molecular crosstalk between the niche and GSCs was deciphered. GSCs maintenance is strongly addicted to several signalling molecules emitted from the niche cells, including Hedgehog (Hh), Wingless (Wg)/Wnt, JAK/STAT and BMP/Dpp-signalling factors, which act in concert to control GSC maintenance^7,11,12^. The determining factor for GSC stemness is the BMP-type ligand Decapentaplegic (Dpp), which is secreted from the somatic niche cells to activate the Dpp signal transducer Mad in the GSC. Activation of Mad occurs by phosphorylation and results in repression of *bag of marbles* (*bam*), which acts in the GSCs daughter, the cystoblast, as the principle differentiation factor. Loss of Dpp entails premature differentiation of GSCs and exhaustion of the germline, i.e. an agametic phenotype, whereas loss of Bam results in tumorous ovaries filled with ever dividing GSCs^6,7,13–15^; for review^5,16^. In addition, a third population of cells, the escort cells, also known as inner germinal sheath cells, is part of the niche-stem cell control system, promoting GSC differentiation as well as maintenance^17–19^.

Besides the finely tuned signalling network originating from the somatic niche microenvironment, several intrinsically acting factors contribute to the balanced homeostasis of germline cell maintenance and differentiation. These include chromatin or histone modifying factors as well as manifold regulators involved in general processes like transcription, mRNA processing, translation, protein modification and stability^20^; for review^21^. Moreover, diverse stress stimuli like starvation or DNA assaults can impact GSC development, shifting the balance between renewal and differentiation into the one or other direction. Protection from DNA-damage, for example through the activity of transposons, is central to germ cells, which are lost upon failure (for overview^21^). Interestingly, several mechanisms underlying germline stem cell loss have been discovered, except the classical apoptotic pathways known from somatic cells^22,23^. GSC loss can cause premature aging of tissues, whereas the accumulation of defective stem cell progeny is connected to cancer stem cell formation and tumour development^24–26^.

Here, we analyze the role of the gene *putzig* (pzg) in cells of germline origin. Pzg encodes a large 160 kDa sized protein that has been identified as integral component of multi-protein complexes, Trf2/Dref and NURF. Whereas Trf2/Dref is involved in the regulation of replication related genes, NURF is essential for chromatin remodelling. Together, Pzg has been shown to play an important role in the regulation of growth and proliferation during *Drosophila* development^27–30^. We already know that *pzg* activity supports homeostasis of somatic cells and tissues during larval development, provoking apoptosis and apoptosis induced compensatory mechanisms when absent^30,31^. Downregulation of *pzg* gene activity in germline cells caused female sterility due to atrophied ovaries, demonstrating the requirement of *pzg* during oogenesis. We provide evidence that loss of *pzg* in germ cell blocks their differentiation and results in cell death within the germarium. Moreover, the levels of growth promoting and regulating factors, predominantly Dpp/Wg and Eiger/JNK signalling, are significantly increased. The induction of growth promoting factors is reminiscent to compensatory effects observed in response to apoptosis in larval somatic cells. Yet, death of germ cells could not be prevented by induction of the anti-apoptotic factors DIAP1 and p35. Due to the very elaborate niche-stem cell signalling circuit in the germarium, ectopic induction of growth promoting and regulating factors mimics a niche like microenvironment, thereby impairing the further differentiation of germ cells. Instead, cell death expands to the whole germarium, perhaps provoked by a forward and feedback loop, resulting in the observed atrophy of *pzg* depleted ovaries. This mechanism may prevent passing erroneous genetic information, caused by the absence of *pzg*, to the next generation.

## Results

**Figure 1:**
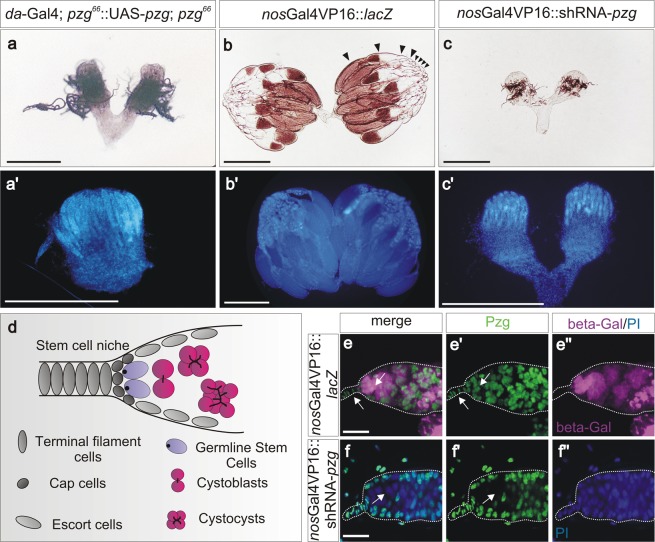
Loss of *pzg* in the germline results in atrophied ovaries. (**a**,**a’**) Rudimentary ovaries are present in *pzg*^*66*^ mutant females, rescued from larval lethality by ubiquitous overexpression of UAS*-pzg* in somatic cells with *da*-Gal4 (*da*-Gal4; *pzg*^*66*^::UASt-*pzg*; *pzg*^*66*^). (**b**,**b’**) In the control (*nos*Gal4VP16::UAS-*lacZ*), ovaries of wild type morphology are observed, with different stages of egg development in the ovarioles (arrowheads). (**c-c’**) Downregulation of *pzg* with shRNA during germ cell development results in small, atrophied ovaries (*nos*Gal4VP16::UAS-shRNA-*pzg*). (**a**–**c**) Phase contrast images. (**a’**–**c’**) DAPI stained tissues; scale bars, 500 μm. (**d**) Sketch of the anterior region of the germarium. The stem cell niche comprises the terminal filament cells and cap cells. Escort cells frame the anterior region of the germarium; they are laterally attached to the Germline Stem Cells (GSCs, blue). GSCs are characterized by the presence of the spherical spectrosome (black dots), whereas developing cysts (magenta) are identified by branched fusomes (black lines). (**e**,**e”**) UAS-*lacZ* visualizes the expression pattern of the maternal *nos*Gal4VP16-driver line in germline cells of the germarium (magenta: anti-beta-Gal); Pzg is in the nuclei of germline- and somatic cells (arrows, green). (**f**,**f”**). Overexpression of UAS-shRNA-*pzg* with *nos*Gal4VP16 leads to depletion of Pzg protein in these germ cells (arrow; green: anti-Pzg; blue: Propidiumiodide (PI) nuclear staining). Scale bars, 10 μm.

*pzg* homozygous mutant animals show severe proliferation and growth defects culminating in early larval death^30^. Continuous overexpression of a *pzg* transgene using the Gal4/UAS system allowed further development of the mutant animals, and a small fraction even reached adulthood without apparent external phenotypes^30^. The females, however, were sterile: they laid no eggs and displayed rudimentary ovaries (Fig. 1a,a’ compare with Fig. 1b,b’). It is well known that the UASt element is not fully active in germ line cells^32^, suggesting an essential function of *pzg* during *Drosophila* oogenesis. Apparently, the UASt-*pzg* transgene was not able to provide sufficient Pzg activity in the female germline, thereby causing ovarian atrophy.

The close proximity to the centromere of the *pzg* locus at 78C5-C6 hampered the generation of FRT79B or FRT80B recombinants for further clonal analysis of *pzg* function in the germ line^33^. Instead, we specifically downregulated *pzg* activity during oogenesis with the help of small hairpin RNAs (shRNA) under UAS control using maternal Gal4-drivers^32,34^. Consistent with the data described above, two different shRNA-*pzg* constructs recapitulated the atrophied ovarian phenotype when induced with different germline specific Gal4-driver lines (Fig. 1c,c’; Fig. S1a–c). We concentrated on one each in the subsequent analyses, shRNA-*pzg* (BL35448) and *nos*Gal4VP16 (BL4937). Efficient and germline-specific downregulation of *pzg* expression was verified *in situ* in adult ovaries, demonstrating that loss of nuclear Pzg protein follows the expression of the maternal Gal4-driver (Fig. 1e,f”). With this tool in hand, we further investigated the function of *pzg* in the female germline of *Drosophila*.

### Depletion of maternal *pzg* activity affects germ cell differentiation

**Figure 2:**
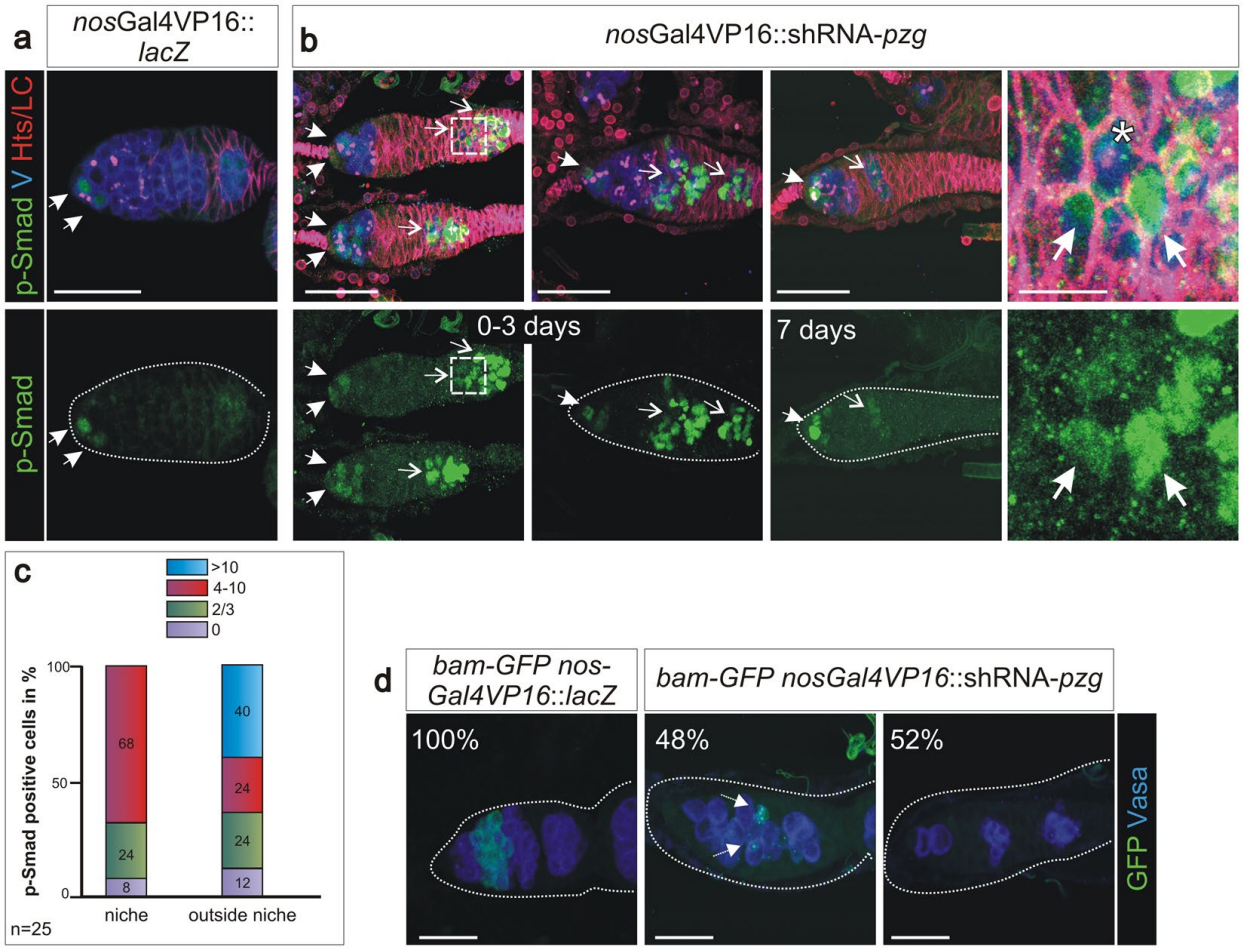
Loss of *pzg* entails a surplus of undifferentiated GSCs. (**a**) In control germaria (*nos*Gal4VP16::UAS-*lacZ*) of 0–3 days on average 2–3 GSCs can be detected by a strong p-Smad signal (green, arrows) and a spherical fusome (red with anti-Hts, appears pink in the overlay) only at the tip of the germarium. Cells of the germline are marked with anti-Vasa (V, blue). (**b**) The number of p-Smad positive cells is strongly elevated in 0–3 days old germaria where *pzg* activity was specifically depleted in cells of the germline (*nos*Gal4VP16::UAS-shRNA-*pzg*). They are localized at next to the niche (closed arrows) and away from the niche (open arrows). Boxed area is magnified in the right panel: note that p-Smad positive cells (green, arrow) co-localize with Vasa (blue). Spectrosome can be discerned in some cells (asterisk; red, appears pink). In one week old germaria, the number of p-Smad positive cells is clearly decreased. (**c)** Quantification of GSCs per germarium in 0–3 days old *nos*Gal4VP16::UAS-shRNA-*pzg* mutants (n = 25). Four categories were formed: no signal 0, 2–3, more (4–10) and >10 as indicated. The left column displays p-Smad positive cells within the niche. Only 24% of *pzg* depleted germaria display two to three p-Smad positive cells within the niche, a value typical for the control, whereas about 70% show more cells. The right column displays p-Smad positive cells outside of the niche. Note that more than half of the germaria show many more p-Smad positive cells mostly outside the niche. (**d**) *bam*-GFP expression (green; anti-GFP) was either absent (52%) or only marginally present (48%) in shRNA*-pzg* depleted germaria (anti-Vasa, blue, n = 25). Scale bars a-d: 25 μm, enlargement 5 μm.

Depletion of *pzg* in germline cells generated ovaries containing only conical, germaria like structures, indicating that subsequent differentiation and development of egg chambers are hampered (Fig. 1c,c’). This may result from the absence of GSCs or from a failure of GSC maintenance and differentiation. GSCs can be identified by the expression of phosphorylated Mad (p-Smad), the activated form of *Drosophila* Smad, as well as by the GSC-specific organelle, the spherical spectrosome^35–37^. Immunostaining of ovaries from young females (0–3 days) for p-Smad reveals typically 2–3 GSCs in the control. In contrast, some of the shRNA-*pzg* depleted germaria contained only 0–1 GSCs within the niche. The majority, however, exhibited in addition a drastic increase in the number of p-Smad positive cells (Fig. 2a–c). Many of them were Vasa positive and contained spherical spectrosomes, typifying them as GSCs (Fig. 2b, see enlargements and Fig. S2). Many of the extra GSCs were no longer restricted to the anterior tip of the germarium, but were detected far posteriorly (Fig. 2b,c). In some cases, the apparent GSC clusters were surrounded by follicle cells giving a cyst-like appearance (Fig. 2b). Overall, loss of *pzg* apparently induced a type of a strongly restricted GSC tumour. The number of p-Smad positive cells decreased with age so that in 7 days old germaria barely any were detected (Fig. 2b).

Since ovaries depleted for Pzg do not develop maturing egg chambers, the GSCs may not carry on with the production of cystoblasts. In order to address the presence of cystoblasts and cystocysts, we studied the expression of *bag of marbles* (*bam)* using a *bam*-GFP reporter^13^. Depletion of Pzg in germline cells resulted in a near complete absence of *bam*-GFP signals. Only marginal and punctual signals were seen in about half of the germaria, whereas no signals were detected in the other half (Fig. 2d). This result is consistent with an elevated Dpp-signalling activity repressing *bam* expression, thereby impairing differentiation of germ cells lacking *pzg*. Apparently, *pzg* activity is required for a proper GSC cell lineage. Because the accumulation of GSCs was the most prominent defect, we focused on this phenotype in our further analyses.

### Depletion of *pzg* activity in the germline increases Dpp-signalling activity

**Figure 3:**
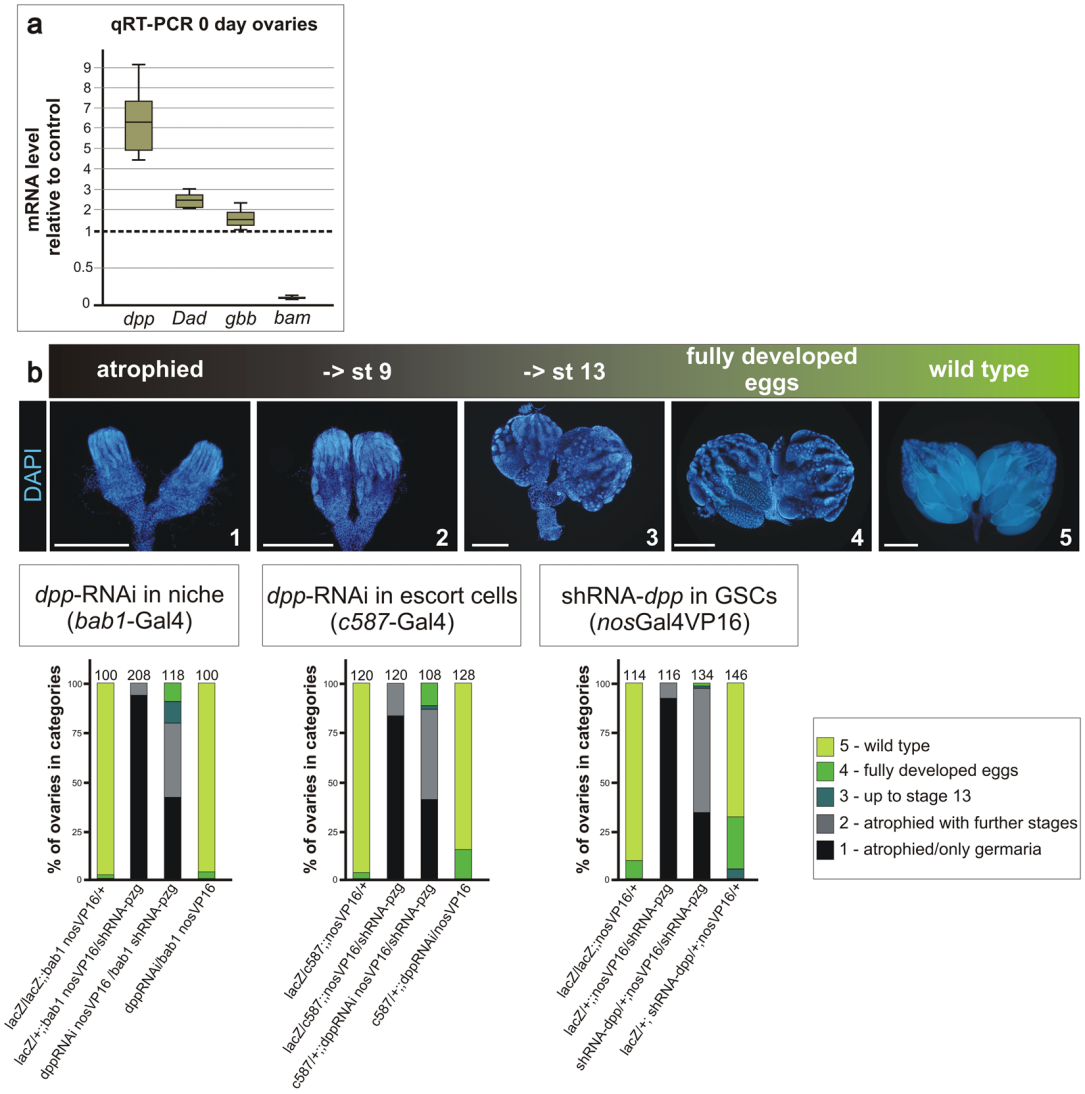
Dpp-signalling activity is de-regulated in *nos*Gal4VP16::UAS*-*shRNA*-pzg* mutant germaria. (**a**) qRT-PCR data gained from ovaries of freshly hatched females: In comparison to the control (*nos*Gal4VP16::UAS-*lacZ*) the level of *dpp*, *gbb* and *Dad* transcripts is increased - whereas *bam* transcripts are strongly reduced in *nos*Gal4VP16::UAS-shRNA-*pzg* depleted ovaries. Four biological and two technical replicates were performed. Three reference genes *dlp, Lamin C* and *slit* were used, and the amplification efficiencies taken into account for determining relative quantities by REST^78^. Median corresponds to expression ratio; mini-max depicts 95% confidence. Expression ratios shown were significant at the level of p < 0.05 using PFRR from REST: p = 0.0280 (*dpp*), p = 0.0595 (*gbb*), p = 0.0160 (*Dad*), p = 0.0200 (*bam*). (**b)** Rescue-assays aiming to deplete *dpp* activity either in the stem cell niche (using *bab1*-Gal4), in escort cells (with *c587*-Gal4) or in the GSC lineage (*nos*Gal4VP16 and UAS-shRNA-*dpp*). The resulting ovaries of 3–5 days old females were subdivided into 5 categories according to their morphology after DAPI staining: **1**: atrophied ovaries, only germaria like structures present; **2**: atrophied ovaries with further developed stages up to stage 9; **3**: development in some ovarioles proceeds up to stage 13; **4**: fully developed eggs can be observed; **5**: wild type ovaries. Scale bars represent 250 μm. Number of analyzed ovaries is given above each bar. Reduction of *dpp* activity in the niche or escort cells rescued the effect of shRNA-*pzg* induction in the germline lineage cells, to a lesser degree also in the germline derived lineage.

As the niche-derived Dpp-signalling activity plays a pivotal role in GSC self-renewal and proliferation, we sought for further evidence of a lost surveillance of BMP/Dpp-signalling activity in shRNA-*pzg* depleted germaria. In order to monitor *dpp* RNA expression levels by quantitative qRT-PCR in shRNA-*pzg* depleted ovaries, RNA was isolated from ovaries derived from freshly eclosed females to minimize the appearance of older egg chamber stages in the reference *nos*Gal4VP16::UAS-*lacZ*. Nevertheless, control ovaries still occasionally harbour further developed egg chambers up to stage 6 (Supplemental Fig. S3a). To minimize effects of developmental stage differences between the shRNA-*pzg* depleted ovaries and control, we took great care in the selection of the reference genes *dlp*, *Lamin C* and *slit*: all three are expressed primarily in the somatic cells of the germarium, i.e. terminal filament cells, niche cells and FSCs^4,38–40^, and average mRNA levels were alike between *pzg* mutant and control (Supplemental Fig. S3b). Consistent with the higher number of p-Smad positive cells *in situ*, *dpp* mRNA levels were about 6-fold increased in shRNA-*pzg* depleted ovaries (Fig. 3a). Furthermore, expression of *glass bottom boat* (*gbb*) mRNA, encoding the second BMP-ligand of the stem cell niche^6^, was also elevated (Fig. 3a). We also assayed two targets of Dpp-signalling activity, *bam* and *Dad*, the former being repressed and the latter activated by Dpp^6,41^. Whereas expression of the Dpp target gene *Dad* was considerably enhanced, *bam* transcript levels were barely detectable (Fig. 3a), in agreement with the faint *bam*-GFP reporter activity in Pzg depleted ovaries (Fig. 2d). Overall, these results suggest that loss of *pzg* activity in germline cells provokes increased Dpp-signalling activity in the germarium, resulting in the retention of GSCs at the expense of the cystoblast daughter cell lineage.

To directly test this hypothesis, we sought to downregulate *dpp* signals in shRNA-*pzg* mutant ovaries. Using specific Gal4-lines, *dpp* mRNA was depleted either in the somatic niche, the somatic escort cells, or within germline cells. Tissue specificity was ensured by using double-stranded RNAi of *dpp* under UAS control for somatic tissue (BL25782), and shRNA-*dpp* for the germline (BL36779). At least 100 ovaries per genotype were stained with DAPI to visualize their morphology and developmental progress within the ovarioles (Fig. 3b). To account for the range of phenotypes, we built five categories according to the developmental progress of egg chamber development. Downregulating *dpp* activity in the local microenvironment of the stem cell niche was achieved with the cap cell specific driver *bric à brac* (*bab1*)-Gal4, which on its own did not affect oogenesis. In contrast, *dpp*-RNAi induction in the cap cells of shRNA-*pzg* mutant ovaries resulted in a strong rescue of the atrophied phenotype: More than 50% of the ovaries contained ovarioles with further developed egg chambers, and in 20% even late egg stages developed (Fig. 3b). A similarly effective improvement was observed by using *c587*-Gal4, which reduced *dpp* levels within the surrounding escort cells^6,36^ (Fig. 3b). Germline specific depletion of *dpp* resulted in a much weaker phenotypic rescue, however, affected oogenesis itself to some degree (Fig. 3b). Together, these results indicate that *pzg* activity is required in the germline to restrict *dpp* levels in order to allow germ cell differentiation to proceed.

### Pzg depletion affects GSC anchoring in the niche

**Figure 4:**
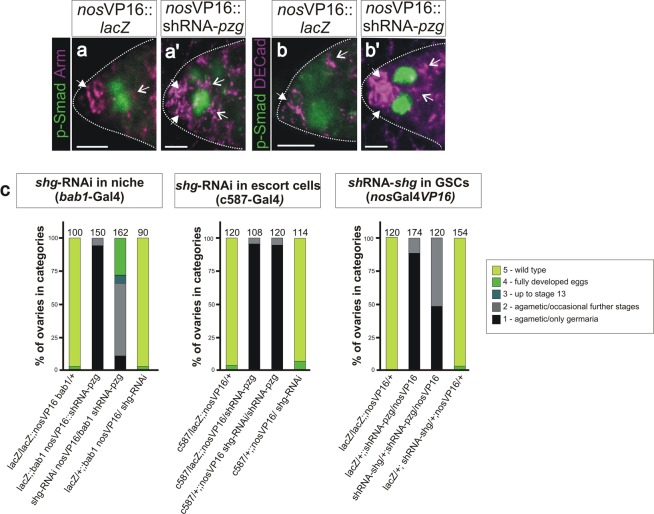
Level of cell adhesion molecules is increased and scattered in shRNA-*pzg* depleted germaria. (**a**,**b’**) Co-staining of p-Smad (green) and Arm (a,a’ magenta) or DE-Cadherin (b,b’ magenta) in 0–3 days old germaria using respective antibodies. In the control (**a**,**b**) *nos*Gal4VP16::UAS-*lacZ*, Arm or DE-Cadherin is mainly detectable at the niche/GSC junction. In *nos*Gal4VP16::UAS-shRNA-*pzg* germaria (**a’**,**b’**), Arm or DE-Cadherin is more enriched at the niche/GSC junction (arrows) and is additionally mislocalized encircling the GSCs (open arrows). Scale bars: 10 μm. (**c**) Rescue assays by changing levels of DE-Cadherin within the GSCs and non-autonomously in cells of the niche (using *bab1*-Gal4) or in escort cells (*c587*-Gal4) in otherwise *pzg* depleted germaria. Reducing the level of *shg* in the niche strongly ameliorates the shRNA-*pzg* atrophied phenotype, whereas concomitant reduction in escort cells (using *c587*-Gal4) did not. Reducing the level of *shg* within the GSC increases the number of atrophied ovaries of category 2. Genotypes of analyzed flies are given below. Categories of rescue are given in the legend. Number of analyzed ovaries is given above the bars.

Our data suggest that loss of *pzg* increases Dpp-signalling activity, enforcing the continuous self-renewal of GSCs and impairing subsequent differentiation. Instead, GSCs agglomerate in the centre of the germarium as apparently undifferentiated GSCs^18^. In a wild type germarium, cell adhesion molecules like DE-Cadherin along the niche/GSC junction contribute to GSC self-renewal and differentiation, by anchoring the GSC within the niche and allowing asymmetric GSC-division to a new GSC and cystoblast daughter (for review^42^). Accordingly, Bam restricts the amount of DE-Cadherin in the differentiating cystoblast^20^. Earlier work has shown that differentiation-defective GSCs can outcompete healthy GSCs from the niche by occupying the limited contacts to the adjacent cap cells^10^. Altered niche contacts may therefore add an explanation for the observed defects in shRNA-*pzg* mutant germaria, like GSCs absence from the niche or ectopic GSCs away from the niche (Fig. 2b,c). In fact, the two cell adhesion molecules, DE-Cadherin and beta-catenin/Armadillo (Arm), showed an abnormal distribution. Whereas, Arm and DE-Cadherin concentrate at the junction between cap cells and GSCs in wild type germaria, both proteins accumulated to higher levels and often framed the p-Smad positive GSCs in shRNA-*pzg* mutant germaria (Fig. 4a,b’). Unbalanced levels of adhesion molecules may provoke a loss of GSCs from the niche proper, allowing their accumulation in the centre of the germarium^42,43^. In this case, we may expect to influence the shRNA-*pzg* mutant phenotype by modulating DE-Cadherin levels at the niche/stem cell junction. To this end, reduction of *shotgun* (*shg*), which encodes DE-Cadherin, was enforced by tissue specific RNAi. Downregulation of *shg* in the cap cells of the niche, but not in the escort cells, considerably improved the atrophied phenotype of shRNA-*pzg* mutant ovaries, allowing well matured egg chambers in about one third of the analyzed females, and even the development of late egg stages in a small fraction (Fig. 4c). Germline-specific downregulation of *shg*, however, had a very mild rescue effect with nearly half of the ovaries being completely atrophied (Fig. 4c). These results further support non-autonomous effects resulting from a loss of *pzg* activity within germline cells acting on somatic cells of the adjacent niche, thereby contributing to the inhibition of further GSC differentiation.

### Loss of *pzg* in germline cells provokes cell death in the germarium

**Figure 5:**
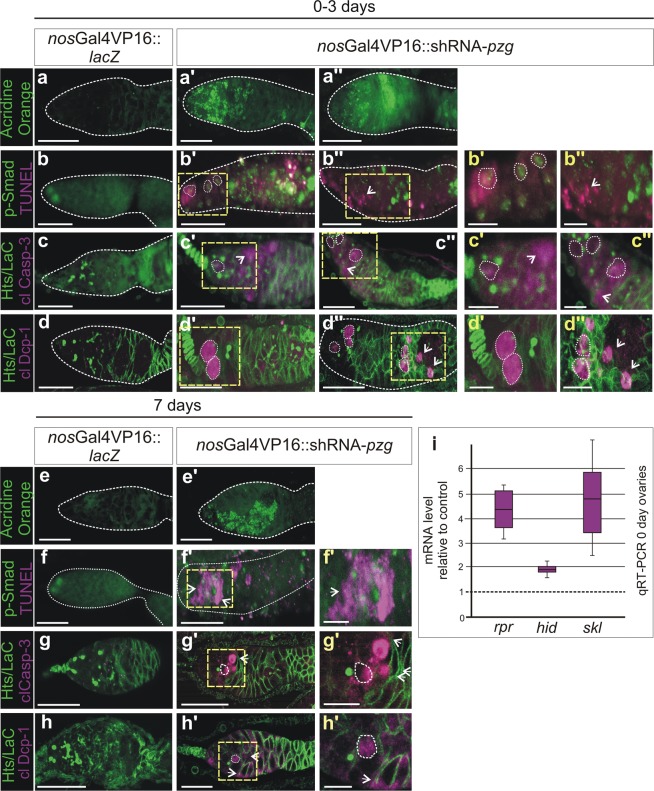
Loss of *pzg* triggers cell death in the germarium. Cell death was assayed in control (*nos*Gal4VP16::UAS-*lacZ*; **a**–**h**) germaria and *pzg*-depleted germaria (*nos*Gal4VP16::UAS-shRNA-*pzg*; **a’**–**h’**), of 0–3 day old females (**a**–**d”**) and of 7d old females (**e**–**h’**). (**a**,**a”**) Acridine Orange dye is selectively retained in dying cells; it is not detected in the control (a) but only in shRNA-*pzg* mutant germaria (**a’,a”**). (**b**,**b”**) TUNEL staining (magenta) marks DNA breaks within dying cells. No TUNEL signal can be detected in the control (b). TUNEL positive cells often co-localize with p-Smad positive cells (green) in the niche (encircled) and further posteriorly, but are also detected next to p-Smad positive cells in shRNA-*pzg* depleted germaria (b”, open arrow). Enlargements of boxed area are show to the right. (**c-c”**) Cleaved Caspase-3 and cleaved Dcp-1 activity (**d-d”**) (both in magenta) were observed only in *pzg* depleted germaria and not in the control. These signals were detected in GSCs labelled with Hts (green, encircled) next to the niche and in cell clusters more posteriorly, as well as in cells not labelled with Hts (open arrows). Co-staining with anti-Hts and Lamin C (green) was used to mark the spectrosomes/fusomes and the outlines of the cells). Enlargements of boxed area are shown to the right. Cell death is still detected in 7 days old germaria from shRNA-*pzg* depleted tissues (**e’–h’**), in contrast to the control (**e–h**). Cell death was detected with Acridine Orange (e-e’, green), TUNEL (f-f’, magenta), cleaved Caspase-3 (g-g’, magenta) and cleaved Dcp-1 (h-h’, magenta). GSCs were marked with p-Smad (f-f’, green), and with Hts (e-h’, green) marking the spectrosome (encircled). The majority of dying cells show no GSC specific marker (arrows). Enlargements of framed area are shown to the right. Scale bars: represent 25 μm, in enlargements 10 μm. (**i)** Quantitative RT-PCR revealed an induction of pro-apoptotic gene expression (*reaper*, *hid* and *sickle*) in shRNA-*pzg* mutant germaria. Four biological and two technical replicates were performed. *Lamin C, dlp* and *slit* served as reference genes. Median corresponds to expression ratio; mini-max depicts 95% confidence. All expression ratios shown were significant at the level of p < 0.05 using PFRR from REST: p = 0.0050 (*rpr*), p = 0.0240 (*hid*), p = 0.0010 (*skl*).

Our results so far imply that a loss of *pzg* activity during germ cell development impairs differentiation by provoking an expanded Dpp-signalling activity and interfering with the adhesion properties of the niche/GSC junction. Both, the altered adhesion as well as increased Dpp activity, are reminiscent of answers from dying cells aiming to maintain tissue homeostasis (for overview^43,44^). Somatic cells undergoing apoptosis emit mitogenic signals, i.e. morphogens like Dpp, to stimulate compensatory proliferation in the surrounding tissue (for overview^44^). In fact, we already know that a loss of *pzg* during wing imaginal development not only induced apoptosis but also compensatory proliferation and apoptosis^31^. We therefore aimed to firmly establish that apoptosis of germline cells occurs as result of *pzg* depletion. We firstly applied Acridine Orange and TUNEL-staining as a hallmark of apoptosis. Indeed, even young (0–3 days) shRNA-*pzg* mutant ovaries strongly retain Acridine Orange dye (Fig. 5a,a”) and displayed several TUNEL-positive cells that were never observed in the control (Fig. 5b,b”). Some of these cells co-stained for p-Smad, whereas others were adjacent to p-Smad positive cells (Fig. 5b’,b”). In addition, the activated form of two different *Drosophila* effector Caspases, cleaved Caspase-3 and Dcp-1, were both observed in shRNA-*pzg* mutant germaria, whereas the control was devoid of specific Caspase activity (Fig. 5c,d”). Not only could we detect Caspase staining within or close to the niche within GSCs, but also in the cluster of undifferentiated GSCs located further posteriorly (Fig. 5c,d”). Caspase activity was not restricted to presumptive GSCs but was also detected in cells nearby (Fig. 5c’,d”). To determine whether pro-apoptotic gene activity of *hid*, *reaper* and *sickle*, one of the first indicators of cell death induction in *Drosophila* somatic cells, is likewise induced, we performed qRT-PCR analyses, which revealed an up to 5-fold induction or pro-apoptotic gene activity (Fig. 5i). In one week old germaria, signs of cell death and Caspase activity were still observed in shRNA-*pzg* mutant germaria, however not in the wild type control (Fig. 5e–h’). The number of dying germ cells, i.e. p-Smad positive cells or cells labelled by a spectrosome, declined, suggesting that they were finally eliminated (Fig. 5f–h’). Overexpression of the somatic Caspase inhibitors DIAP1 and/or p35 within the cells of the germline only slightly ameliorated the atrophied phenotype, allowing the development of a higher percentage of further developed egg stages up to stage 9 (Fig. S4). Unexpectedly, Acridine Orange and TUNEL staining as well as Dcp-1 Caspase activity were still observed in these germaria (Fig. S5), indicating that DIAP1/p35 overexpression cannot suppress cell death in the germ line. Either, the two may not be functional inhibitors of apoptosis and Caspase activity in the female germline and/or the observed cell death effects are not only based on apoptotic death but include other non-apoptotic mechanisms of cell death.

### Loss of *pzg* in germline cells triggers the expression of growth promoting and regulating genes

**Figure 6:**
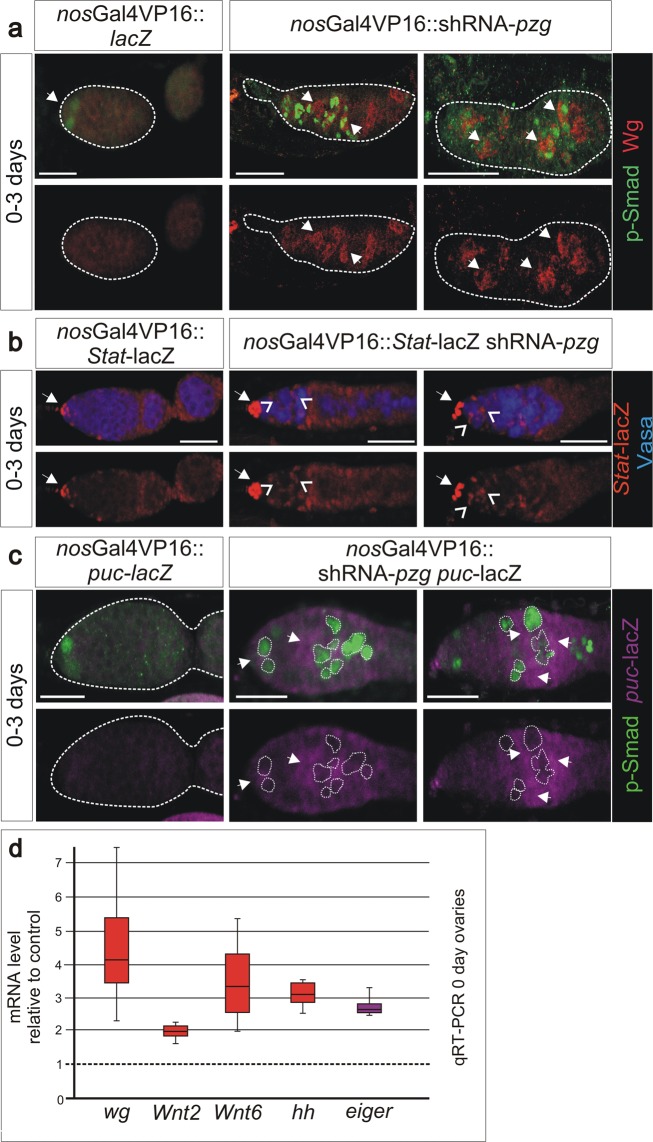
Loss of *pzg* results in higher levels of growth regulatory factors. (**a**) Wg staining (red) is barely detectable in the control germaria (encircled; left panel); two GSCs (anti-pSmad, green, arrows) are located at the niche. In shRNA-*pzg* depleted germaria (right panels), Wg protein accumulates at higher levels and encircles the ectopic GSCs (stained with anti-pSmad, green, arrows). (**b**) *Stat* activity was detected with a *Stat*-lacZ reporter (anti-beta-Gal, red) in the cap cells of the niche (arrow) in control and *pzg* depleted germaria. Loss of *pzg* activity in the GSCs results in an increase in *Stat*-lacZ signals in the niche. Moreover, *Stat*-lacZ activity is detected also in cells surrounding the GSCs (open arrowheads. (**c**) In comparison to the control, *puc*-lacZ reporter activity (magenta, anti-beta-Gal) is increased in shRNA-*pzg* depleted germaria (arrows), notably framing p-Smad positive cells (green). Scale bars: 25 μm in all panels. (**d**) qRT-PCR revealed a considerable accumulation of *wg*, *Wnt*2, *Wnt6*, *hh* or *eiger* transcripts in *nos*Gal4VP16::UAS-shRNA-*pzg* mutant germaria in comparison to the control (*nos*Gal4VP16::UAS-*lacZ*). Four biological and two technical replicates were performed. Amplification efficiencies of the three reference genes *dlp, Lamin C* and *slit* were taken into account for determining relative quantities by REST. Median corresponds to expression ratio; mini-max depicts 95% confidence. All expression ratios shown were significant at the level of p < 0.05 using PFRR from REST: p = 0.0060 (*wg*), p = 0.0150 (*Wnt2*), p = 0.0190 (*Wnt6*), p = 0.0210 (*hh*), p = 0.0125 (*eiger*).

As shown above, shRNA-*pzg* mutant germ cells undergo cell death with typical signs of apoptosis. As a consequence, the dying germline cells may emit survival signals acting non-autonomously onto the surrounding cells. According to this working hypothesis, the observed rise in *dpp* and *Dad* expression (Fig. 3a), as well as the compromised adhesion properties (Fig. 4a,b’), might be a direct consequence of cell death induced by *pzg* depletion. In this case, we might expect the release of additional morphogens from the dying cells, as has been described for somatic tissue (for overview^44,45^). Indeed, immunostaining revealed a strong enrichment of Wg protein in cell groups that were in direct contact to the p-Smad positive cells, an effect never observed in the control (Fig. 6a). In accord with this, mRNA expression of *wg* and several other *Wnt* ligands known to promote proliferation in the germarium^19,46^ was elevated in *pzg* depleted ovaries as well (Fig. 6d). Likewise, we found significantly higher expression levels for *hedgehog* (*hh*) and *Stat*-lacZ (Fig. 6b,d). Moreover, RNAi-mediated depletion of Stat in either somatic niche cells or germline cells, occasionally allowed development of further developed egg chambers (Fig. S4). Inhibiting *wg*-signalling, particularly by overexpressing Axin (Axn) in escort cells, also improved the development of *pzg* depleted ovaries (Fig. S4). These rescue effects were not as strong as those observed by the reduction of *dpp* in the niche or in escort cells, emphasizing the central role of a deregulated Dpp-signalling as causative for the *pzg*-mediated ovarian atrophy.

**Figure 7:**
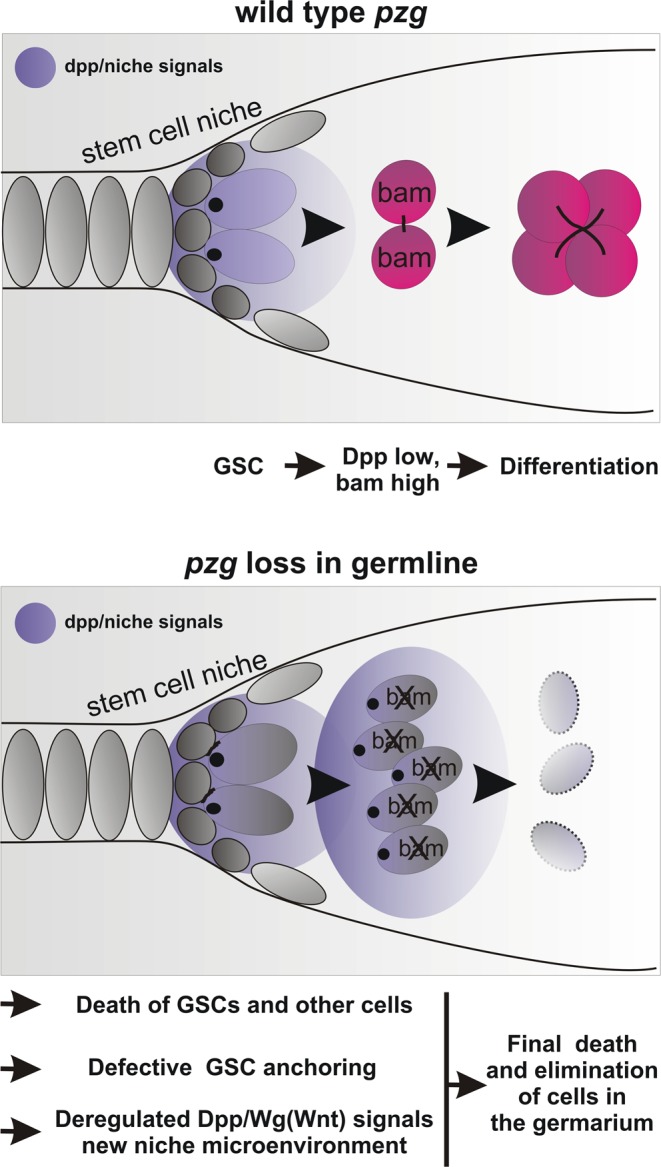
Model of *pzg* function in the germline. In a wild type ovary, short range Dpp-signalling activity is confined to the stem cell niche. Upon division, the daughter cell moves away from the niche thereby ensuring the upregulation of the differentiation gene *bam*. When *pzg* is depleted in germline cells, cellular homeostasis and stem cell niche communication is disturbed: As a consequence, cell death and growth promoting and regulating factors are induced. This provokes a new niche-like microenvironment inhibiting further differentiation of GSCs and finally the elimination of the undifferentiated, accumulated GSCs within the germarium.

Overall, our results suggest that *pzg* depleted germline cells cannot differentiate but instead produce cell death-mediated mitogenic signals. Although these signals are important for the division and maintenance of GSCs in the female germline, we observed GSC loss with time. Perhaps the dying GSCs not only emit growth promoting but also other signals, which contribute to the final death of the cells in the germarium. Such a process is known from apoptotic somatic cells and relies on the production of the TNF orthologue Eiger by apoptotic cells^47^. Interestingly, we observed a significant induction of *eiger* expression by qRT-PCR in shRNA-*pzg* mutant ovaries (Fig. 6d). Moreover, an increase in JNK-signalling readout, visualized with *puc*-lacZ, was observed predominantly in cells surrounding p-Smad positive cells (Fig. 6c). As JNK-signalling is involved in various aspects of apoptosis including phagocytosis during oogenesis (for overview^48^), these results suggest induction of cell death in the germarium induced by Eiger and JNK-signalling activity as a consequence of *pzg* depletion. Together with the growth regulators induced as well, these two might contribute to the disturbed balance between proliferation- and differentiation in a *pzg* depleted germline (Fig. 7).

## Discussion

Normal development strictly depends on programmed cell death for sculpting organs and tissues. This also applies to *Drosophila* oogenesis: notably the oocyte-supporting nurse cells undergo apoptosis at the end of egg maturation (for review^49^). Moreover, diverse stress or insults may lead to premature death of egg chambers or the contained oocytes. For example, egg chambers may degenerate in response to bad nutritional conditions, chemical insults or altered hormonal signalling, thereby avoiding the unnecessary investment of energy into egg production^24,50^; for review^49^. Although the elimination of the cells is undisputed, the underlying molecular processes are debated. Both, Caspase dependent and independent processes have been described, which even share several components in their molecular transduction machinery^22,51,52^.

The case appears different for stem cells. Several adult stem cell types in *Drosophila* as well as in other organisms manage to avoid cell death in response to severe DNA damage, thereby resisting genotoxic agents^23,53–55^. Similarly, ovarian GSCs from *Drosophila* are protected against stress-provoked apoptosis through joint efforts from multiple signalling pathways, combining intrinsically acting survival factors with anti-apoptotic signals from neighbouring cells. Here, the receptor tyrosine kinase Tie/Tie-2 acts as a gatekeeper for apoptosis inhibition in the GSCs by activating *bantam*-mediated inhibition of pro-apoptotic gene activity^23^. Our study adds *pzg* to the collection of genes involved in GSCs biology and survival.

### Loss of *pzg* in the germline: The pros and cons for apoptosis and apoptosis related processes

In the absence of *pzg*, germ cells display many typical characteristics of programmed cell death: Firstly, they exhibit typical apoptotic markers like fragmented DNA labelled by TUNEL, internal pH imbalance detected by Acridine Orange staining, and most importantly the accumulation of activated Caspases Caspase-3 and Dcp-1. Secondly, transcription of pro-apoptotic genes *reaper, hid* and *sickle*, is markedly increased in *pzg* depleted germaria. Moreover, the elevation of JNK-signalling readout as well as higher expression levels of the TNF *eiger* are indicators of cell death, eventually eliminating the germline cells in the absence of *pzg*. Finally, while accumulating cell adhesion proteins DE-Cadherin and Armadillo, GSCs can be displaced from the stem cell niche. Apart from signs of cell death, enhanced production of morphogenic, proliferative signals was observed a well, notably of Dpp, Wg/Wnt/Hh and Stat. These effects are reminiscent of Apoptosis-induced Proliferation (AiP) and Apoptosis-induced Apoptosis (AiA), well known from somatic tissues. Compensatory cell proliferation elicited by apoptotic cells in epithelia is well described in *Drosophila* and other organisms (for review^44,45^). By emitting proliferative signals, dying cells actively engage with their surroundings aiming to perpetuate the cellular homeostasis and finally survival of the organism. Similarly, loss of *pzg* activity in larval wing discs results in programmed cell death accompanied by the apoptosis-induced proliferation and apoptosis^31^. In the context of early oogenesis, however, cell death responses appear to have devastating consequences (see model in Fig. 7). Albeit germ cells gain the propensity to renew by the release of morphogens, notably Dpp continuously precludes GSCs differentiation but not division, culminating in the accumulation of undifferentiated GSC-like cells. In other words, dying GSCs might release signals that mimic the microenvironment of the stem cell niche, enforcing germ cell self-renewal at the expense of differentiation. The differentiation block effectively brings oogenesis to a halt (Fig. 7). Moreover, as overexpression of Dpp elicits the death of ovarian somatic cells by the upregulation of pro-apoptotic genes^56^, a feedback and forward loop may amplify the apoptotic response. Accordingly, interception at the level of Dpp most convincingly ameliorated the atrophied ovary phenotype.

Although we observed a strong activity of the effector Caspases Dcp-1 and Caspase-3, and in addition DNA breaks by TUNEL staining, a concomitant induction of anti-apoptotic factors p35 and DIAP1 in cells of the germline caused a minor rescue of the shRNA-*pzg* mutant ovary phenotype. Although counter-intuitive at first, the result may be expected for the caspase inhibitor p35^57^: by inhibiting effector caspase activity, but not the induction either of pro-apoptotic genes or initiator caspases, p35 provokes the so-called ‘undead’ cells that still release proliferation signals. Consequently, hyperplastic tumorous overgrowth is induced^58,59^. As outlined above, release of proliferation signals in the germarium trigger a differentiation-block, halting oogenesis. Accordingly, p35 may rescue from cell death on the one hand and enhance the phenotypic consequences of excessive Dpp on the other hand. In contrast to p35, DIAP1 inhibits the activity of the initiator caspase Dronc in *Drosophila*, antagonized by the physical binding of pro-apoptotic proteins^60–62^; for review^63^. The strong activation of *rpr* and *hid* resulting from *pzg* depletion may hence outcompete DIAP1 activity. Likewise, DIAP1 overexpression did also not suffice to block apoptosis during disc regeneration^64^. Moreover, as DIAP1 acts as a positive regulator of Wg/Wnt-signalling independent of its anti-apoptotic role^65^, it might instead fuel the *pzg*-mediated self-renewal loop. DIAP1 activity requires Ubiquitin-binding co-factors to regulate Caspase activity, which might be lacking or not functional in the female germline^66,67^. Accordingly, DIAP1 overexpression in the germline had little effect on the apoptotic markers observed in response to *pzg* depletion (Fig. S5) or on the resultant ovarian atrophy (Fig. S4). Therefore other anti-apoptotic proteins, apart from DIAP1, might be important to combat apoptotic cell death in the female germline. Interestingly, upregulation of DIAP1 in spermatogonia is not sufficient to rescue cell death, which was explained by a combination of apoptotic and non-canonical apoptotic mechanisms occurring in this tissue^52,68^. Apparently, apoptotic cell death and also non-apoptotic cell death is observed after *pzg* depletion in the germline.

### The role of *pzg* in the female germline

Although *pzg* loss is associated with the death of somatic and germline derived cells, the mechanisms mediating these processes seem to differ. In somatic cells, the loss of *pzg* activity mainly triggers release of Wg but not of Dpp^31^, whereas the loss of *pzg* in germ cells results in a very strong induction of Dpp activity, which seem to propel the germline defects. Several recent studies have shown, that Dpp activity in the female germline is regulated by multiple intrinsic and extrinsic mechanisms (for review^16,17^). Transcriptional activation of *dpp* is mediated by JAK/STAT- signalling^14,15^, and restricted by Engrailed^69^. Moreover, several epigenetic regulators as well as chromatin modifying factors influence Dpp-signalling activity, thereby participating in the control of germline stem cell differentiation (for review^21^). As Pzg is known to be an integral part of the nucleosome remodelling NURF complex^29^, it is conceivable that *pzg* might exert its influence on GSC development via NURF. However, mutations in NURF complex components affect GSC self-renewal due to a precocious differentiation and reduced Dpp-signalling level in females and males, which contradict our observations^70–72^. By contrast, maternal depletion of the transcriptional regulator DREF, another well-known interactor of Pzg^27,28^, caused atrophied ovaries, as revealed by a large-scale RNAi screen in *Drosophila* female stem cells^73^. DREF and Pzg cooperate during cell and tissue homeostasis in larval tissues^28^, and may so during oogenesis as well.

Pzg is intimately involved in germ cell survival and loss of *pzg* activity in the germline provokes a pronounced cell death response. As *pzg* mutants show signs of telomere instability and therefore increased genomic instability^74^, GSCs devoid of *pzg* activity should not be passed to the next generation. We identified Dpp activation as the principle factor in the molecular response resulting from *pzg* loss. This is intriguingly different from that of the soma, where Wg plays the dominant role. Pzg presumably co-operates with further, yet unidentified factors in the germline. The exact molecular mechanisms of Pzg protective activity in germline stem cells, including its interaction partners, are of great interest in the future.

## Materials and Methods

### Fly strains, developmental conditions and sample size

Flies were cultured on standard fly food at 18 °C. Experiments were performed on uncrowded cultures using 15 virgins and 10 males per cross, changed every other day on ‘enriched food’ (10 g agar, 0.5 g CaCl_2_, 60 g glucose, 20 g yeast extract, 0.5 g MgSO_4_, 20 g peptone, 30 g sucrose, 80 g dry yeast and 6 ml propionic acid, per liter) at 25 °C.

For antibody staining, ovaries were dissected from 0–3 or 7 days old females. Ovarioles from up to 20 females were prepared for each experiment and visualized by fluorescence microscopy. A minimum of 10 representative germaria was documented per experiment. All experiments were performed at least in triplicate.

Fly stocks were combined and recombined by standard genetics, and confirmed molecularly with single fly PCR.

Information on fly strains is available on https://flybase.org. ***Germline specific Gal4-driver lines:**** nos*Gal4VP16 4937-Gal4 (BL4937), *nos*Gal4VP16 UAS-GFP*-alpha Tub84B* 7253-Gal4 (BL*7253*), *MTD*-Gal4 (Maternal Triple driver) (BL31777). ***Somatic Gal4-driver lines:**** bab1*-Gal4/TM3 *Sb*^1^ (BL6802), *c587*-Gal4 (BL67747), *da*-Gal4^75^. ***UAS-shRNA-lines:*** UAS-shRNA-*dpp* (BL36779), UAS-shRNA-*pzg* (BL35448), UAS-shRNA-*pzg*/CyO (BL57793), UAS-shRNA-*shg*/CyO (BL38207), UAS-shRNA-*Stat92E*/CyO (BL35600). ***UAS-RNAi-lines:*** UAS-*dpp*-RNAi (BL25782), UAS-*shg*-RNAi (BL27689), UAS-*Stat92E*-RNAi (BL31318). ***UAS-lines***: UAS-*Axn*GFP/TM3Sb (BL7225), UASp-*p35*/CyO-actin (G2154)^76^, UASp-*DIAP1* (BL63819), UASp-*lacZ*^32^, UAS-*pzg*^30^. ***Reporter lines:**** bam*-GFP/CyO^13^, *Stat*-lacZ/TM3, *Sb*^1^ *Ser*^1^ (BL11681), *puc*-lacZ^77^. ***Mutant strains:**** pzg*^*66*^/TM6B, *Tb*^1^ Ubi-GFP^30^, *wg*^*CX4*^/CyO (gift from K. Basler, Zürich, Switzerland).

### Immunochemistry

#### Staining of adult ovaries

Ovaries were fixed in 4% paraformaldehyde for 15 min, washed 3–4 times with PBX (PBS + 0.3% Triton X-100), and blocked for at least 30 min in PBX and 4% normal goat serum at room temperature prior to antibody incubation which was done overnight at 8 °C. After several washes with 1x PBX and pre-incubation with 4% NGS for at least 30 minutes, secondary antibodies were added and incubated either overnight at 8 °C or for 2 hours at room temperature. Primary antibodies used were: guinea-pig anti-Putzig^28^ (anti-Pzg; 1:1000), mouse anti-Armadillo (anti-Arm N27A1; 1:20 – E. Wieschaus), mouse anti-beta-galactosidase (anti-beta-Gal; 40–1a; 1:50 - J.R. Sanes), mouse anti-Hts (anti-1B1; 1:20 – H.D. Lipshitz), mouse anti-Lamin C (anti-LC28.26; 1:20 – P.A. Fisher), mouse anti-Wingless (anti-4D4; 1:50 – S.M. Cohen), rat anti-DE-Cadherin (anti-DCAD2; 1:50 – T. Uemura), rat anti-Vasa (1:50 – A.C. Spradling) (developed by the investigators mentioned, obtained from DSHB, Iowa, USA), mouse anti-GFP (1:50, Santa Cruz Biotechnology, USA, sc-9996), rabbit anti-cleaved Caspase-3 (#9661) and rabbit anti-cleaved Dcp-1 (#9578) (both 1:200, Cell Signaling, USA), rabbit anti-phospho Smad 1/5 (anti-pSmad; 1:50, Cell Signaling, Germany; #9516). Goat secondary antibodies with minimal cross-reactivity coupled to FITC, Cy3 or Cy5 were from Jackson Immuno-Research Laboratories (Dianova, Hamburg; Germany). Ovaries were mounted in VectaShield (Vector Laboratories, California, USA), and analyzed with a Bio-Rad MRC1024 confocal system coupled to a Zeiss Axiophot microscope (Carl Zeiss AG, Oberkochen, Germany) using LaserSharp 2000 imaging software. Pictures were assembled with Corel-PhotoPaint and CorelDRAW Version 9.0 software.

#### Acridine Orange staining of ovaries

Ovaries were dissected in EBR buffer (130 mM NaCl, 4.7 mM KCl, 1.9 mM CaCl_2_, 10 mM Hepes pH 6.9) and incubated in 1.6 μM Acridine Orange in 0.1 M sodium phosphate buffer, pH 7.2 for 3 min in the dark. Ovarioles were mounted in Vectashield (Vector Laboratories, California, USA) and documented as above. The elapsed time from dissection to the end of documentation did not exceed 20 min.

#### Terminal deoxynucleotidyl transferase dUTP nick end labeling (TUNEL) staining

Dying cells were detected with the *In Situ Cell Death Detection Kit* TMR Red (Roche, Mannheim, Germany) according to Wang and Page-Mc Caw^19^. Briefly, ovaries were fixed in 4% paraformaldehyde for 20 min, washed several times in PBS, followed by incubation with PBX (PBS, 0.1% Triton X-100, 0.1% sodium citrate). 100 μl of the TUNEL reaction mixture was added, followed by incubation in the dark at 37 °C for one hour. After several washes in PBX (PBS, 0.1% Triton X-100), ovaries were further co-stained with other antibodies and processed accordingly.

#### Quantitative RT-PCR

Quantitative RT-PCR was performed on four biological and two technical replicates of each genotype with 25 ovaries from freshly hatched females. Poly(A)^+^ RNA was extracted with the *Dynabeads*^*TM*^ *mRNA DIRECT*^*TM*^ *Purification Kit* (Invitrogen, Thermo Fisher Scientific, Waltham, USA) and treated with DNaseI (New England Biolabs, Frankfurt, Germany) to remove remaining DNA. 30–75 ng mRNA was reverse transcribed with *qScriber cDNA Synthesis Kit* (highQu, Kraichtal, Germany). Real time qPCR was performed with *Blue S’Green qPCR Kit* (Biozym, Hessisch-Oldendorf, Germany) on 1 μl cDNA (0.15–2.4 ng) in 10 μl end volume using MIC magnetic induction cycler (bms, Pots Point, Australia), always including target and no-template controls: a hot start (95 °C 2 min) and 40 cycles of 95 °C 5 s/68 °C 10 s was followed by a melt curve analysis (72–95 °C at 0.3°/s) to select for specific amplification. Absence of DNA was tested in a non-RT control for every sample. Three different reference genes (*dlp*, *Lamin C* and *slit*) were selected based on their expression predominantly in the somatic cells of the germarium^4,38–40^, and based on similar expression levels in mutant and control tissue determined by Δct values (Fig. S3b). Relative quantification of the data was performed with micPCR software Version 2.8 based on REST^78^, taking target efficiency into account. REST uses Pairwise Fixed Reallocation Randomization Test (PFRR) for statistical evaluation. Expression values p < 0.05 are considered to be statistically significant. The following primer pair sequences (in parentheses) are listed at DRSC FlyPrimer bank^79^: *bam* (PD70062), *Dad* (PA60210), *dpp* (PP5962), *eiger* (PD70026), *gbb* (PD70018), *hh* (PP25933), *rpr* (PD41945), *skl* (PD45510), *wg* (PP23467), *wnt2* (PP31089), *wnt6* (PP33695). Other primers used: *dlp* (Upper, 5′ ATG CGG GTA GTG GAG CTG GTT CT 3′; Lower, 5′ GTC CGG CTT ATG GCT GGG TTC 3′), *hid* (Upper 5′ CGA AGG CCG AGA AGA AGA AAC CAC 3′; Lower 5′ TTC ATC GCG CCG CAA AGA AG 3′), *Lamin C* (Upper 5′ CTG GAG GAA CCT CTT GGA CAC GGA 3′; Lower 5′ CCT ACC GCA CAG CAG TTT GTC 3′); *slit* (Upper 5′ CAC CAT AGG GCG CGA CAT CG 3′; Lower 5′ TCG AAT CCC CCT TGT TGT ACT ACC A 3′),

#### Rescue assays and DAPI staining of adult ovaries

For rescue assays, molecularly verified flies were crossed and incubated at 25 °C; food was changed every 2–3 days. Newly hatched females were selected and analyzed at day 3–5. To this end, adult ovaries were fixed in 4% paraformaldehyde for 15 min, washed 3–4 times with PBX, followed by DAPI staining (2.5 mg/ml in PBT) for 4 min. After several washes with PBX, ovaries were mounted in 80% glycerol to be documented with an Axioskop 2 plus microscope (Carl Zeiss Microscopy GmbH, Oberkochen, Germany) connected to a Canon EOS 700 camera (Canon, Tokyo, Japan).

## Supplementary information

Supplementary Information

## Data Availability

All data supporting the findings of this study are available within the article and the Supplementary Information Files or from the corresponding author upon request.

## References

[1] Bastock R, St Johnston D (2008). Drosophila oogenesis. Curr Biol.

[2] Spradling AC (1993). Germline Cysts: Communes that work. Cell.

[3] Li L, Xie T (2005). Stem cell niche: structure and function. Annu Rev Cell Dev Biol.

[4] Xie T, Spradling AC (2000). A niche maintaining germ line stem cells in the *Drosophila* ovary. Science.

[5] Kirilly D, Xie T (2007). The *Drosophila* ovary: an active stem cell community. Cell Research.

[6] Song X (2004). Bmp signals from niche cells directly repress transcription of the differentiation-promoting gene, *bag of marbles*, in germline stem cells in the *Drosophila* ovary. Development.

[7] Xie T, Spradling AC (1998). *decapentaplegic* is essential for the maintenance and division of germline stem cells in the *Drosophila* ovary. Cell.

[8] Song X, Zhu CH, Doan C, Xie. T (2002). Germline stem cells anchored by adherens junctions in the *Drosophila* ovary niches. Science.

[9] Song X, Xie T (2002). DE-cadherin-mediated cell adhesion is essential for maintaining somatic stem cells in the *Drosophila* ovary. Proc Natl Acad Sci USA.

[10] Jin Z (2008). Differentiation-defective stem cells outcompete normal stem cells for niche occupancy in the *Drosophila* ovary. Cell Stem Cell.

[11] Losick VP, Morris LX, Fox DT, Spradling A (2011). *Drosophila* stem cell niches: a decade of discovery suggests a unified view of stem cell regeneration. Dev Cell.

[12] Xie T (2013). Control of germline stem cell self-renewal and differentiation in the *Drosophila* ovary: concerted actions of niche signals and intrinsic factors. WIREs Dev Biol.

[13] Chen D, McKearin D (2003). Dpp signaling silences *bam* transcription directly to establish asymmetric divisions of germline stem cells. Curr Biol..

[14] Lopez-Onieva L, Fernandez-Minan A, Gonzalez-Reyes A (2008). Jak/Stat signaling in niche support cells regulates *dpp* transcription to control germline stem cell maintenance in the *Drosophila* ovary. Development.

[15] Wang L, Li Z, Cai Y (2008). The JAK/STAT pathway positively regulates DPP signaling in the *Drosophila* germline stem cell niche. J Cell Biol.

[16] Harris RE, Ashe HL (2011). Cease and desist: modulating short-range Dpp signalling in the stem-cell niche. EMBO Rep..

[17] Chen S, Wang S, Xie T (2011). Restricting self-renewal signals within the stem cell niche: multiple levels of control. Curr. Opin Genet Dev.

[18] Kirilly D, Wang S, Xie T (2011). Self-maintained escort cells form a germline stem cell differentiation niche. Development.

[19] Wang X, Page-Mc Caw A (2018). Wnt6 maintains anterior escort cells as an integral component of the germline stem cell niche. Development.

[20] Shen R, Wang C, Yu J, Xie T (2009). elF4A controls germline stem cell self-renewal by directly inhibiting BAM function in the *Drosophila* ovary. Proc Natl. Acad. Sci. USA.

[21] Gleason RJ, Anand A, Kai T, Chen X (2018). Protecting and diversifying the germline. Genetics.

[22] Bakhrat A, Pritchett T, Peretz G, McCall K, Abdu U (2010). *Drosophila* Chk2 and p53 proteins induce stage-specific cell death independently during oogenesis. Apoptosis.

[23] Xing Y, Su TT, Ruohola-Baker H (2015). *Tie*-mediated signal from apoptotic cells protects stem cells in *Drosophila melanogaster*. Nature Communications.

[24] Drummond-Barbosa D, Spradling AC (2001). Stem cells and their progeny respond to nutritional changes during *Drosophila* oogenesis. Dev Biol..

[25] Ma X (2016). DNA damage-induced Lok/Chk2 activation compromises germline stem cell self-renewal and lineage differentiation. Development.

[26] Rosen JM, Jordan CT (2009). The increasing complexity of the cancer stem cell paradigm. Science.

[27] Hochheimer A, Zhou S, Zheng S, Holmes MC, Tjian R (2002). Trf2 associates with DREF and directs promoter-selective gene expression on *Drosophila*. Nature.

[28] Kugler SJ, Nagel AC (2007). *putzig* is required for cell proliferation and regulates Notch activity in *Drosophila*. Mol Biol Cell.

[29] Kugler SJ, Nagel AC (2010). A novel Pzg-NURF complex regulates Notch target activity. Mol Biol Cell.

[30] Kugler SJ, Gehring EM, Wallkamm V, Krüger V, Nagel AC (2011). The Putzig-NURF nucleosome remodeling complex is required for ecdysone receptor signaling and innate immunity in *Drosophila melanogaster*. Genetics.

[31] Zimmermann M, Kugler SJ, Schulz A, Nagel AC (2015). Loss of *putzig* activity results in apoptosis during wing imaginal development in *Drosophila*. PLoS ONE.

[32] Rørth P (1998). Gal4 in the female germline. Mech Dev.

[33] Chou TB, Perrimon N (1992). Use of a yeast-site specific recombinase to produce female germline chimeras in *Drosophila*. Genetics.

[34] Ni JQ (2011). A genome-scale shRNA resource for transgenic RNAi in *Drosophila*. Nat Methods.

[35] Gilboa L, Forbes A, Tazuke SI, Fuller MT, Lehmann R (2003). Germ line stem cell differentiation in *Drosophila* requires gap junctions and proceeds via an intermediate state. Development.

[36] Kai T, Spradling AC (2003). An empty *Drosophila* stem cell niche reactivated the proliferation of ectopic cells. Proc. Natl Acad. Sci. USA.

[37] Lin H, Yue L, Spradling AC (1994). The *Drosophila* fusome, a germline-specific organelle, contains membrane skeletal proteins and functions in cyst formation. Development.

[38] Hartman TR (2015). Novel tools for genetic manipulation of follicle stem cells in the Drosophila ovary reveal an integrin-dependent transition from quiescence to proliferation. Genetics.

[39] Hayashi Y (2012). Glypicans regulate JAK/STAT signaling and distribution of the Unpaired morphogen. Development.

[40] Song X, Xie T (2003). Wingless signaling regulates the maintenance of ovarian somatic cells in *Drosophila*. Development.

[41] Casanueva MO, Ferguson EL (2004). Germline stem cell number in the *Drosophila* ovary is regulated by redundant mechanisms that control Dpp signaling. Development.

[42] Chen S, Lewallen M, Xie T (2013). Adhesion in the stem cell niche: biological roles and regulation. Development.

[43] Monier B (2015). Apico-basal forces exerted by apoptotic cells drive epithelium folding. Nature.

[44] Fogarty CE, Bergmann A (2017). Killers creating new life: caspases drive apoptosis-induced proliferation in tissue repair and disease. Cell Death Differ.

[45] Martín FA, Pérez-Garijo A, Morata G (2009). Apoptosis in *Drosophila*: compensatory proliferation and undead cells. Int. J. Dev. Biol..

[46] Wang X, Page-Mc Caw A (2014). A matrix metalloproteinase mediates long-distance attenuation of stem cell proliferation. J Cell Biol.

[47] Pérez-Garijo. A, Fuchs Y, Steller H (2013). Apoptotic cells can induce non-autonomous apoptosis through the TNF pathway. Elife.

[48] Serizier SB, McCall K (2017). Scrambled eggs: Apoptotic cell clearance by non-professional phagocytes in the *Drosophila* ovary. Front Immunol.

[49] McCall K (2004). Eggs over easy: cell death in the *Drosophila* ovary. Dev Biol.

[50] Buszczak M, Cooley L (2000). Eggs to die for: cell death during *Drosophila* oogenesis. Cell Death Differ.

[51] Kutscher LM, Shaham S (2017). Non-apoptotic cell death in animal development. Cell Death Differ.

[52] Yacobi-Sharon K, Namdar Y, Arama E (2013). Alternative germ cell death pathway in *Drosophila* involves HtrA2/Omi, lysosomes, and a caspase-9 counterpart. Dev Cell.

[53] Blanpain C, Mohrin M, Sotiropoulou PA, Passegué E (2011). DNA-damage response in tissue-specific and cancer stem cells. Cell Stem Cell.

[54] Reya T, Morrison SJ, Clarke MF, Weissman IL (2001). Stem cells, cancer, and cancer stem cells. Nature.

[55] Wang X (2011). Histone H3K9 Trimethylase Eggless controls germline stem cell maintenance and differentiation. PLoS Genet.

[56] Kang I (2018). Identification of target genes regulated by the *Drosophila* histone methyltransferase Eggless reveals a role of Decapentaplegic in apoptotic signaling. Sci Reports.

[57] Hay B, Wolff T, Rubin GM (1994). Expression of baculovirus P35 prevents cell death in *Drosophila*. Development.

[58] Pérez-Garijo A, Shlevkov E, Morata G (2009). The role of Dpp and Wg in compensatory proliferation and in the formation of hyperplastic overgrowths caused by apoptotic cells in the *Drosophila* wing disc. Development.

[59] Ryoo HD, Gorenc T, Steller H (2004). Apoptotic cells can induce compensatory cell proliferation through the JNK and the Wingless signaling pathways. Dev Cell.

[60] Chai J (2003). Molecular mechanism of Reaper-Grim-Hid-mediated suppression of DIAP1-dependent Dronc ubiquitination. Nat. Struct. Biol..

[61] Wu JW, Cocina AE, Chai J, Hay BA, Shi Y (2001). Structural analysis of a functional DIAP1 fragment bound to Grim and Hid peptides. Mol Cell.

[62] Yan N, Wu JW, Chai J, Li W, Shi Y (2004). Molecular mechanisms of DrICE inhibition by DIAP1 and removal of inhibition by Reaper, Hid and Grim. Nat. Struct. Mol. Biol.

[63] Fuchs Y, Steller H (2011). Programmed cell death in animal development and disease. Cell.

[64] Diaz-Garcia S, Ahmed S, Baonza A (2016). Analysis of the function of apoptosis during imaginal wing disc regeneration in *Drosophila melanogaster*. PLoS ONE.

[65] Hanson AJ (2012). XIAP monoubiquitylates Groucho/TLE to promote canonical Wnt signaling. Mol. Cell.

[66] Lee T (2011). *Drosophila* IAP-1-mediated ubiquitylation controls activation of the initiator caspase DRONC independent of protein degradation. PLoS Genetics.

[67] Huang Q (2014). Ubr3 E3 ligase regulates apoptosis by controlling the activity of DIAP1 in *Drosophila*. Cell Death and Differentiation.

[68] Hasan S, Hétié P, Matunis EL (2015). Niche signaling promotes stem cell survival in the *Drosophila* testis via the JAK-STAT target DIAP1. Developmental Biology.

[69] Luo L, Siah CK, Cai Y (2017). Engrailed acts with Nejire to control *decapentaplegic* expression in the *Drosophila* ovarian stem cell niche. Development.

[70] Ables ET, Drummond-Barbosa D (2010). The steroid hormone ecdysone functions with intrinsic chromatin remodeling factors to control female germline stem cells in *Drosophila*. Cell Stem Cell.

[71] Cherry CM, Matunis EL (2010). Epigenetic regulation of stem cell maintenance in the *Drosophila* testis via the nucleosome-remodeling factor NURF. Cell Stem Cell.

[72] Xi R, Xie T (2005). Stem cell self-renewal controlled by chromatin remodeling factors. Science.

[73] Yan D (2014). A regulatory network of *Drosophila* germline stem cell self-renewal. Dev Cell.

[74] Silva-Sousa R, López-Panadès E, Piñeyro D, Casacuberta E (2012). The chromosomal proteins JIL-1 and Z4/Putzig regulate the telomeric chromatin in *Drosophila melanogaster*. PLoS Genet.

[75] Wodarz A, Hinz U, Engelbert M, Knust E (1995). Expression of crumbs confers apical character on plasma membrane domains of ectodermal epithelia of *Drosophila*. Cell.

[76] Werz C (2005). Mis-specified cells die by an active gene-directed process, and inhibition of this death results in cell fate transformation in *Drosophila*. Development.

[77] Martín-Blanco E (1998). Puckered encodes a phosphatase that mediates a feedback loop regulating JNK activity during dorsal closure in *Drosophila*. Genes Dev.

[78] Pfaffl MW, Horgan GW, Dempfle L (2002). Relative expression software tool (REST) for a group-wise comparison and statistical analyses of relative expression results in real-time PCR. Nucl Acids Res.

[79] Hu Y (2013). FlyPrimerBank: an online database for *Drosophila melanogaster* gene expression analysis and knockdown evaluation of RNAi reagents. G3 (Bethesda).

