# Supplementary material for: Loss of putzig in the germline impedes germ cell development by inducing cell death and new niche like microenvironments
Source: Sci Rep. 2019 Jun 24;9:9108. doi: 10.1038/s41598-019-45655-5 (PMC6591254; doi:10.1038/s41598-019-45655-5)
Supplement: Supplementary file 1 — Supplementary Information [file 41598_2019_45655_MOESM1_ESM.pdf]

Supplementary Information

Loss of *putzig* in the germline impedes germ cell development by inducing cell death and new niche like microenvironments

Ludmilla Kober[1¶], Mirjam Zimmermann[1¶], Michaela Kurz[1], Melanie Bayer[1], Anja C. Nagel[1*]

Content:

Supplementary Figure 1

Supplementary Figure 2

Supplementary Figure 3

Supplementary Figure 4

Supplementary Figure 5

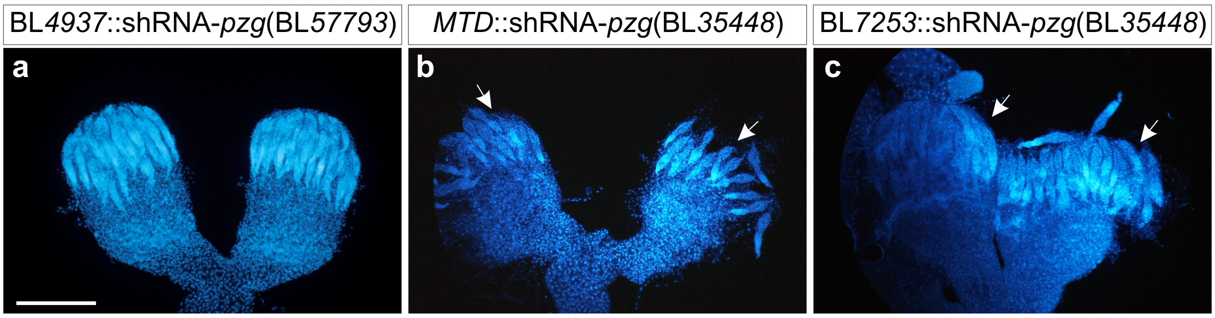

Supplementary Figure 1. shRNA-*pzg* depletion in the germline lineage results in atrophied ovaries

A second UAS-shRNA-*pzg* line and two additional germline specific Gal4-driver lines were used to confirm the observation of atrophied ovaries resulting from depletion of *pzg* activity. (**a**) UAS-shRNA-*pzg* line (BL*57793*) also provokes rudimentary ovaries when induced in germline cells (*nos*Gal4VP16::UAS-shRNA-*pzg*). **(b-c)** The UAS-shRNA-*pzg* line (BL*35448)* used throughout this work, results in atrophied ovaries as well when induced with either *MTD*-Gal4 (**b**) or BL*7253*-Gal4 (**c**) driver lines. Arrows point to rudimentary germaria-like structures. Ovaries were stained with DAPI. Scale bar represents 200 μm.

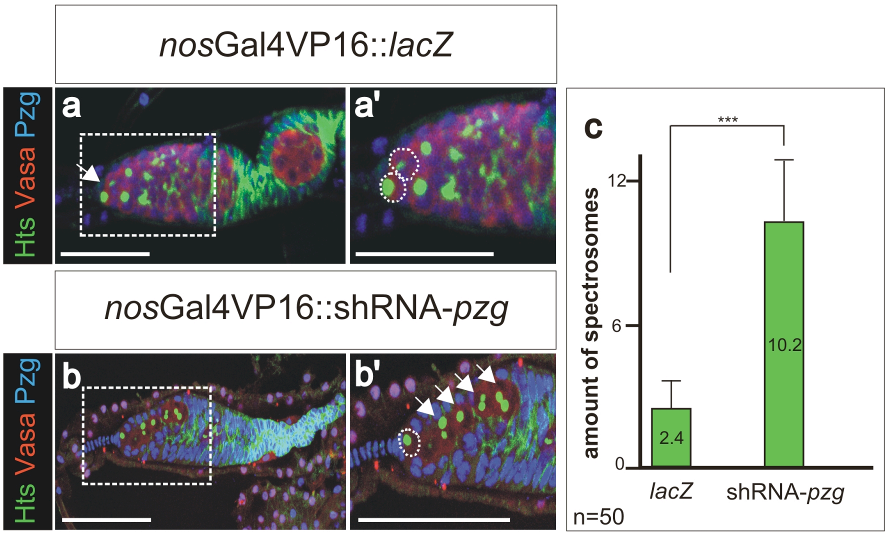

Supplementary Figure 2. Number of spectrosome containing cells is increased in shRNA-*pzg* mutant germaria

(**a**) Control germaria (*nos*Gal4VP16::UAS-*lacZ*) contain 2-3 GSCs with a spherical spectrosome (arrow). (**a'**) Enlargement of framed region in (a). GSCs are encircled. (**b**) The number of spectrosome containing cells is increased in *nos*Gal4VP16::UAS-shRNA-*pzg* depleted germaria. (**b'**) Enlargement of framed region in (b). GSC in the niche is encircled. Anti-Hts (green) detects spectrosomes and fusomes of differentiating GSCs, anti-Vasa (red) the germ cells and anti-Pzg (blue) marks nuclei of cells. Note localization of additional Hts positive spectrosomes in cells lacking nuclear Pzg signals, presumably corresponding to undifferentiated GSCs (arrows). Scale bars represent 25 µm. (**c**) Quantification of cells with round spectrosomes in 0-3 days old germaria (n=50 germaria). Error bars represent standard deviation (SD). Significance was analyzed using unpaired Student's t-Test. ***, $p < 0.001$.

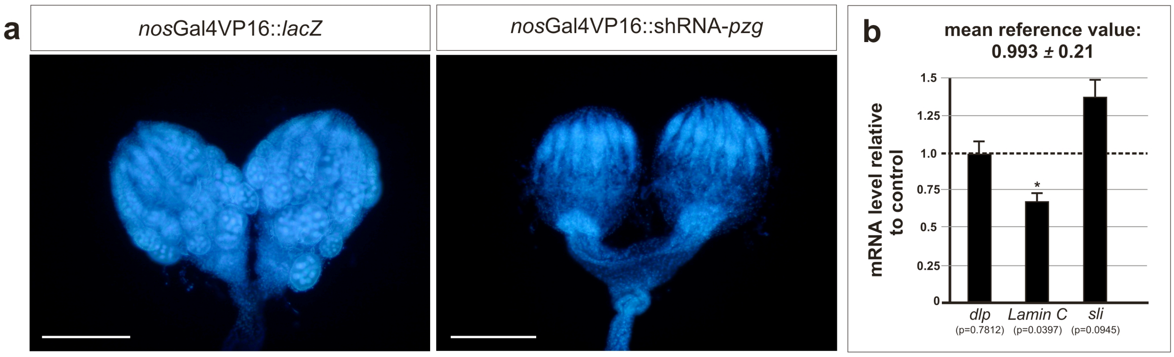

Supplementary Figure 3. Expression ratio of reference genes

(**a**) DAPI stained ovaries from freshly hatched females of the control (*nos*Gal4VP16::UAS-*lacZ*) and *nos*Gal4VP16::UAS-shRNA-*pzg*. Note egg chambers up to stage 6 in the control, never observed in *pzg* depleted ovaries. Scale bars represent 200 μm. (**b**) Transcript abundance of reference genes *dlp*, *Lamin C* and *sli* was determined by Δct in mRNA from 25 ovaries of control and shRNA-*pzg* depleted ovaries. Expression levels are similar; mean value for the three genes is 0.993 ± 0.21. Data are from four biological and two technical replicates. SEM is shown. Significance was tested by a two-tailed student t-test analysis relative to *nos*Gal4VP16::UAS-*lacZ*; p-values are given below the genes.

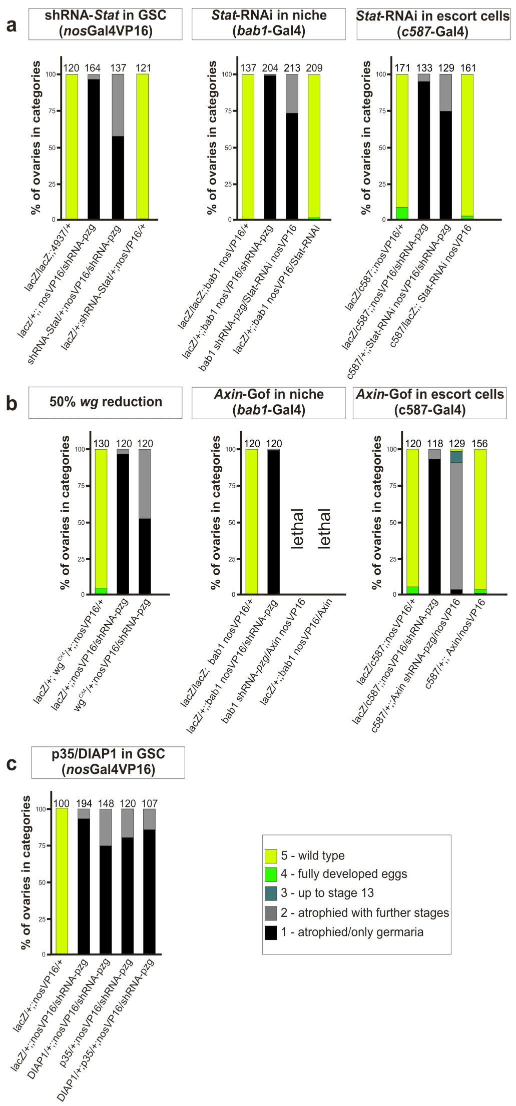

Supplementary Figure 4. Rescue-assays with *Stat*, *wg* and anti-apoptotic factors *p35* and *DIAP1*

(**a**) Reducing the activity of *Stat* autonomously in the GSC-lineage or non-autonomously in the niche or escort cells increased the percentage of females with ovaries of category 2. (**b**) In a heterozygous wg^{CX4} mutant background the percentage of females with atrophied ovaries harbouring further stages increased to app. 50%. Repression of *wg*-signalling activity using UASt-Axin in escort cells (c587-Gal4) allows the development of further egg stages up to stage 9, whereas *bab1*-Gal4 mediated induction is lethal. (**c**) Concomitant induction of anti-apoptotic factors p35, DIAP1 or both together slightly increased the fraction of further developed stages when induced in GSCs. 95-213 ovaries were analyzed per genotype as indicated. Genotypes are given below the bars.

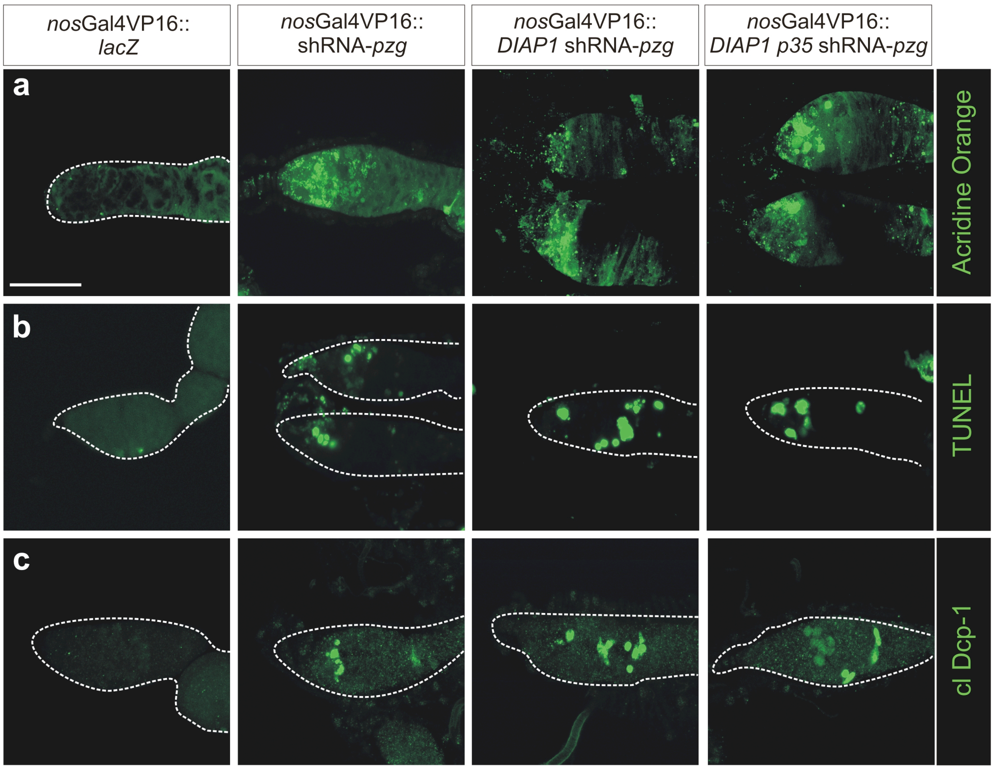

Supplementary Figure 5. Cell death stainings

Even after overexpression of *p35* and/or *DIAP1*, cell death is observed in shRNA-*pzg* depleted ovaries. 0-3 days old ovaries were stained with (**a**) Acridine Orange (**b**) TUNEL or (**c**) anti-cleaved Dcp-1. Scale bar, 25 µm for all panels. Genotypes analyzed: *nos*Gal4VP16::UAS-*lacZ*, *nos*Gal4VP16::UAS-shRNA-*pzg*, *nos*Gal4VP16::UAS-*DIAP1;;* UAS-shRNA-*pzg, nos*Gal4VP16:: UAS-*DIAP1;*UAS-*p35;* UAS-shRNA-*pzg*.
